# Excitotoxic glutamate causes neuronal insulin resistance by inhibiting insulin receptor/Akt/mTOR pathway

**DOI:** 10.1186/s13041-019-0533-5

**Published:** 2019-12-19

**Authors:** Igor Pomytkin, Irina Krasil’nikova, Zanda Bakaeva, Alexander Surin, Vsevolod Pinelis

**Affiliations:** 1 Institute of Regenerative Medicine, I.M. Sechenov First Moscow State Medical University, Trubetskaya Street, 8, 119991 Moscow, Russia; 2 Scientific Center for Biomedical Technologies, Federal Medical and Biological Agency, 143442 Svetlye Gory, Moscow region, Russia; 3 National Medical Research Center for Children’s Health, Russian Ministry of Health, Lomonosov’s prospect, 2, 119991 Moscow, Russia; 4 The Institute of General Pathology and Pathophysiology, Baltiyskaya Street, 8, 125315 Moscow, Russia

**Keywords:** Insulin, Glutamate excitotoxicity, Central insulin resistance

## Abstract

**Aim:**

An impaired biological response to insulin in the brain, known as central insulin resistance, was identified during stroke and traumatic brain injury, for which glutamate excitotoxicity is a common pathogenic factor. The exact molecular link between excitotoxicity and central insulin resistance remains unclear. To explore this issue, the present study aimed to investigate the effects of glutamate-evoked increases in intracellular free Ca^2+^ concentrations [Ca^2+^]_i_ and mitochondrial depolarisations, two key factors associated with excitotoxicity, on the insulin-induced activation of the insulin receptor (IR) and components of the Akt/ mammalian target of rapamycin (mTOR) pathway in primary cultures of rat cortical neurons.

**Methods:**

Changes in [Ca^2+^]_i_ and mitochondrial inner membrane potentials (ΔΨ_m_) were monitored in rat cultured cortical neurons, using the fluorescent indicators Fura-FF and Rhodamine 123, respectively. The levels of active, phosphorylated signalling molecules associated with the IR/Akt/mTOR pathway were measured with the multiplex fluorescent immunoassay.

**Results:**

When significant mitochondrial depolarisations occurred due to glutamate-evoked massive influxes of Ca^2+^ into the cells, insulin induced 48% less activation of the IR (assessed by IR tyrosine phosphorylation, pY^1150/1151^), 72% less activation of Akt (assessed by Akt serine phosphorylation, pS^473^), 44% less activation of mTOR (assessed by mTOR pS^2448^), and 38% less inhibition of glycogen synthase kinase β (GSK3β) (assessed by GSK3β pS^9^) compared with respective controls. These results suggested that excitotoxic glutamate inhibits signalling via the IR/Akt/mTOR pathway at multiple levels, including the IR, resulting in the development of acute neuronal insulin resistance within minutes, as an early pathological event associated with excitotoxicity.

## Main text

An acute impairment in the biological response to insulin in the brain, known as central insulin resistance, was identified during stroke [1] and traumatic brain injury [2], for which excitotoxicity, which is caused by excessive glutamate release, is a key pathogenic factor [3].

The exact molecular link between insulin resistance and glutamate excitotoxicity remains unclear. Based on published data, an abnormal rise in intracellular free Ca^2+^ concentration ([Ca^2+^]_i_) and decreased mitochondrial inner membrane potential (ΔΨ_m_) are factors associated with the excitotoxic glutamate [4–6], which could potentially affect insulin signalling. The presence of Ca^2+^ (1 mM) has been shown to reduce the insulin-induced tyrosine phosphorylation of the insulin receptor (IR) in hippocampal synaptic preparations [7]. Glutamate has been shown to reduce the tyrosine phosphorylation of the IR when added after the prolonged insulin-mediated stimulation of hippocampal neuronal cultures [8]. Protonophore-induced decreases in ΔΨ_m_ have been shown to evoke concomitant decreases in the IR tyrosine phosphorylation in response to insulin, indicating that mitochondrial depolarisation is an independent causative factor for neuronal insulin resistance [9]. However, the effects of glutamate-evoked changes in [Ca^2+^]_i_ and ΔΨ_m_ on the insulin-induced activation of the IR/Akt/mammalian target of rapamycin (mTOR) and glycogen synthase kinase (GSK)3β pathways have never been studied. Here, we investigated whether excitotoxic glutamate affects the insulin-induced phosphorylation of IR/Akt/mTOR pathway components during significant mitochondrial depolarisations caused by the massive influx of Ca^2+^ into the cells.

**Fig. 1:**
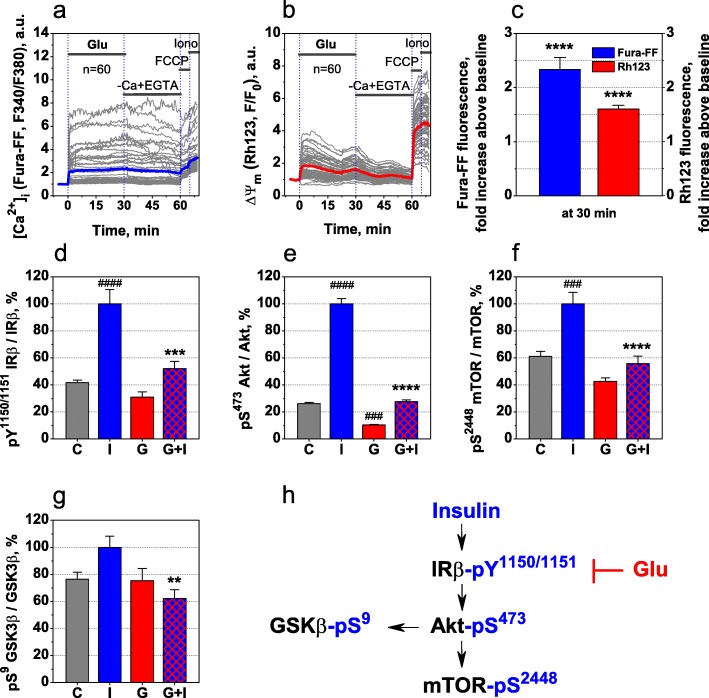
Excitotoxic glutamate inhibits IR/Akt/mTOR pathway **a** Dynamics of [Ca^2+^]_i_ and **b** ΔΨ_m_ in single rat cortical neurons, loaded simultaneously with Fura-FF and Rh123 dyes and exposed to 100 μM glutamate. Grey lines represent sixty single neurons. Blue and red lines represent the respective means of [Ca^2+^]_i_ and ΔΨ_m_, averaged across sixty individual neurons at every time point. **c** Fura-FF and Rh123 fluorescence 30 min after the onset of glutamate exposure, expressed as the fold increase over baseline (at − 5 min). Data are the mean ± SEM from sixty neurons. ****P < 0.0001 compared to respective baselines (one-way ANOVA with repeated measures, followed by Tukey’s post hoc test). **d-g** Levels of **d** IRβ pY^1150/1151^, **e** Akt pS^473^, **f** mTOR pS^2448^, and **g** GSK3β pS^9^ in rat cortical neurons exposed to 0 nM (C) or 100 nM insulin for 15 min (I), 100 μM glutamate for 30 min **g**, or sequentially to 100 μM glutamate for 30 min and 100 nM insulin for 15 min (G + I). Bar graphs represent the levels of the phosphoproteins, normalised against respective total protein levels, in cell lysates and expressed as a percentage of levels in insulin-treated cells (group I). Each value represents the mean ± SEM from six independent cultures (cell populations obtained from twelve separate rats, two per culture). ^###^*P* < 0.001, ^####^*P* < 0.0001 compared with untreated control **c**; **P < 0.01, ***P < 0.001, ****P < 0.0001 compared with insulin **i** (one-way ANOVA, followed by Tukey’s post hoc test for multiple comparisons). **h** Scheme illustrating the inhibitory effects of glutamate on the insulin-induced activation of the IR/Akt/mTOR pathway.

To determine the times during which glutamate induces significant mitochondrial depolarisation, rat cortical neurons were exposed to 100 μM glutamate and changes in [Ca^2+^]_i_, and ΔΨ_m_ were monitored for 30 min. Detailed methods for the preparation of primary cortical neuronal cultures and measuring [Ca^2+^]_i_ and ΔΨ_m_ are described in Additional file 1. As expected, glutamate evoked a rapid increase in [Ca^2+^]_i_, followed by a decreased in ΔΨ_m_ in the cells (Fig. 1a and b, Additional file 2: Tables S3 and S4). After 30 min of glutamate exposure, the mean [Ca^2+^]_i_ level increased by 2.4-fold above baseline (F_148,8732_ = 44.8, *P* < 0.0001), and the mean ΔΨ_m_ value significantly decreased by 1.6-fold below baseline (F_148,8732_ = 182.8, *P* < 0.0001) (Fig. 1a–c, one-way analysis of variance [ANOVA] with repeated measures, followed by Tukey’s post hoc test). Therefore, the 30-min interval for glutamate exposure was selected for subsequent experiments.

Next, we investigated the effects of glutamate on the insulin-induced activation of signalling molecules in the IR/Akt/mTOR and GSK3β pathways. Rat cortical neuronal cultures were sequentially exposed to either 100 μM excitotoxic glutamate concentration or no glutamate for 30 min, then stimulated with 100 nM insulin for 15 min and lysed. The levels of the tyrosine-phosphorylated IR β-subunit (IRβ pY^1150/1151^), the serine-phosphorylated Ser/Thr kinase Akt (Akt pS^473^), the serine-phosphorylated mammalian target of rapamycin (mTOR pS^2448^), and the serine-phosphorylated glycogen synthase kinase 3β (GSK3β pS^9^) were measured in the cell lysates and normalised against the total levels of these proteins, as outlined in Additional file 1. A one-way ANOVA analysis revealed a significant difference between groups for IRβ pY^1150/1151^ (Fig. 1d, F_3,20_ = 23.62, *P* < 0.0001), Akt pS^473^ (Fig. 1e, F_3,20_ = 388.6, *P* < 0.0001), mTOR pS^2448^ (Fig. 1f, F_3,20_ = 19.69, *P* < 0.0001), and GSK3β pS^9^ (Fig. 1g, F_3,20_ = 4.48, *P* = 0.0146). Tukey’s multiple comparisons test showed that insulin stimulation resulted in a significant increase in IRβ pY^1150/1151^ (Fig. 1d, *P* < 0.0001), Akt pS^473^ (Fig. 1e, *P* < 0.0001), and mTOR pS^2448^ (Fig. 1f, *P* < 0.001) levels compared with their respective controls (Additional file 1: Tables S1 and S2). In the glutamate pre-treated neurons, insulin induced 48% less IR phosphorylation at pY^1150/1151^ (Fig. 1d, *P* < 0.001), 72% less Akt phosphorylation at pS^473^ (Fig. 1e, *P* < 0.0001), 44% less mTOR phosphorylation at pS^2448^ (Fig. 1f, *P* < 0.0001), and 38% less GSK3β phosphorylation at pS^9^ (Fig. 1g, *P* < 0.01) compared with non-glutamate treated controls. Neuronal IR becomes fully active following autophosphorylation at Y^1150/1151^ [10], which triggers downstream signalling via the Akt/mTOR and GSK3β pathways. The serine-phosphorylation of Akt at S^473^ [11] and mTOR at S^2448^ [12] are crucial for their activation, whereas the serine-phosphorylation of GSK3β at S^9^ [13] leads to its inhibition. Therefore, our results suggested that excitotoxic glutamate inhibits insulin-induced IR activation and the downstream IR/Akt/mTOR signalling pathway (Fig. 1h).

The primary finding of the present study was that excitotoxic glutamate inhibits the IR/Akt/mTOR pathway, resulting in the development of acute neuronal insulin resistance during periods of significant mitochondrial depolarisation caused by glutamate-evoked massive influxes of Ca^2+^. This rapid loss of neuronal insulin sensitivity appears to be one of the earliest pathological events associated with glutamate excitotoxicity. These results are in complete agreement with our previous findings that mitochondria control IR autophosphorylation in neurons and that mitochondrial depolarisation causes the loss of insulin sensitivity during the IR autophosphorylation stage [9, 14]. Recently we showed that pre-treatment with insulin prevents the glutamate-evoked increases in [Ca^2+^]_i_ and decreases in ΔΨ_m_, protecting rat cortical neurons against excitotoxicity [15]. The glutamate effect and the protective effects of insulin were both completely abrogated by MK 801, an inhibitor of Ca^2+^ influx, via the N-methyl-D-aspartate (NMDA) receptor and the plasmalemmal Na^+^/Ca^2+^ exchanger operating in reverse mode [15]. Collectively, these findings suggested that the modulation of intracellular Ca^2+^ levels plays a critical role in negative cross-talk between insulin and glutamate signalling during excitotoxicity. Glutamate induces an increase in [Ca^2+^]_i_ and a decrease in ΔΨ_m_, which inhibit IR activation. In turn, insulin prevents the glutamate-evoked rise in [Ca^2+^]_i_ and mitochondrial depolarisation, protecting against excitotoxicity.

In conclusion, this study showed that glutamate excitotoxicity is causative for central insulin resistance and may induce the acute loss of insulin signalling within minutes under mitochondrial depolarisation conditions. Therefore, the use of agents designed to prevent mitochondrial depolarisation may be a reasonable approach to the treatment of acute neuronal insulin resistance during excitotoxicity.

## Supplementary information

**Additional file 1: Figure S1.** Representative images of fluorescence staining of cell culture on (A) DAPI, (B) GFAP, (C) bIIITubulin, and (D) Merge, scalebar 100 μm. (A), **Table S1.** Raw data of phosphoproteins measurements in cell lysates, **Table S2.** Results of statistical analysis.

**Additional file 2: Table S3.** Raw data of [Ca2+]i (Fura-FF f340/f380 fluorescence) measurements.

## Data Availability

All data are available in the Additional file 1 and Additional file 2.

## Abbreviations

[Ca^2+^]_i_: Intracellular free Ca^2+^ concentration
Akt: The Ser/Thr kinase Akt
ANOVA: analysis of variance
GSK3β: The glycogen synthase kinase 3β
IR: Insulin receptor
IRβ: Insulin receptor β-subunit
mTOR: The mammalian target of rapamycin
ΔΨ_m_: Mitochondrial inner membrane potential

## References

[1] Lai TW, Zhang S, Wang YT (2014). Excitotoxicity and stroke: identifying novel targets for neuroprotection. Prog Neurobiol.

[2] Karelina K, Sarac B, Freeman LM, Gaier KR, Weil ZM (2016). Traumatic brain injury and obesity induce persistent central insulin resistance. Eur J Neurosci.

[3] Guerriero RM, Giza CC, Rotenberg A (2015). Glutamate and GABA imbalance following traumatic brain injury. Curr Neurol Neurosci Rep.

[4] Nicholls DG (2004). Mitochondrial dysfunction and glutamate excitotoxicity studied in primary neuronal cultures. Curr Mol Med.

[5] Abramov AY, Duchen MR (1777). Mechanisms underlying the loss of mitochondrial membrane potential in glutamate excitotoxicity. Biochim Biophys Acta.

[6] Duchen MR, Surin A, Jacobson J (2003). Imaging mitochondrial function in intact cells. Methods Enzymol.

[7] Zhao W, Chen H, Xu H, Moore E, Meiri N, Quon MJ, Alkon DL (1999). Brain insulin receptors and spatial memory. Correlated changes in gene expression, tyrosine phosphorylation, and signaling molecules in the hippocampus of water maze trained rats. J Biol Chem.

[8] Zhao WQ, De Felice FG, Fernandez S, Chen H, Lambert MP, Quon MJ (2008). Amyloid beta oligomers induce impairment of neuronal insulin receptors. FASEB J.

[9] Persiyantseva NA, Storozhevykh TP, Senilova YE, Gorbacheva LR, Pinelis VG, Pomytkin IA (2013). Mitochondrial H2O2 as an enable signal for triggering autophosphorylation of insulin receptor in neurons. J Mol Signal.

[10] White MF, Kahn CR (1994). The insulin signaling system. J Biol Chem.

[11] Alessi DR, Andjelkovic M, Caudwell B, Cron P, Morrice N, Cohen P (1996). Mechanism of activation of protein kinase B by insulin and IGF-1. EMBO J.

[12] Nave BT, Ouwens M, Withers DJ, Alessi DR, Shepherd PR (1999). Mammalian target of rapamycin is a direct target for protein kinase B: identification of a convergence point for opposing effects of insulin and amino-acid deficiency on protein translation. Biochem J.

[13] Cross DA, Alessi DR, Cohen P, Andjelkovich M, Hemmings BA (1995). Inhibition of glycogen synthase kinase-3 by insulin mediated by protein kinase B. Nat.

[14] Storozhevykh TP, Senilova YE, Persiyantseva NA, Pinelis VG, Pomytkin IA (2007). Mitochondrial respiratory chain is involved in insulin-stimulated hydrogen peroxide production and plays an integral role in insulin receptor autophosphorylation in neurons. BMC Neurosci.

[15] Krasil'nikova I, Surin A, Sorokina E, Fisenko A, Boyarkin D, Balyasin M, Demchenko A, Pomytkin I, Pinelis V (2019). Insulin protects cortical neurons against glutamate excitotoxicity. Front Neurosci.

