# Supplementary material for: Excitotoxic glutamate causes neuronal insulin resistance by inhibiting insulin receptor/Akt/mTOR pathway
Source: Mol Brain. 2019 Dec 19;12:112. doi: 10.1186/s13041-019-0533-5 (PMC6923972; doi:10.1186/s13041-019-0533-5)
Supplement: Supplementary file 1 — Additional file 1: Figure S1. Representative images of fluorescence staining of cell culture on (A) DAPI, (B) GFAP, (C) bIIITubulin, and (D) Merge, scalebar 100 μm. (A), Table S1. Raw data of phosphoproteins measurements in cell lysates, Table S2. Results of statistical analysis. [file 13041_2019_533_MOESM1_ESM.docx]

Additional file 1

**Excitotoxic glutamate causes neuronal insulin resistance by inhibiting insulin receptor/Akt/mTOR pathway**

Igor Pomytkin*, Irina Krasil’nikova, Zanda Bakaeva, Alexander Surin, Vsevolod Pinelis.

*Corresponding author. E-mail: ipomytkin@mail.ru

**This file includes:**

Materials and Methods

Figure 1S

Table S1

Table S2

**Materials and Methods**

**Primary culture of rat cortical neurons**

Experiments with animals were performed in accordance with the ethical principles and regulatory documents recommended by the European Convention on the Protection of Vertebrate Animals used for experiments (Guide for the Animals and Eighth Edition. 2010), as well as in accordance with the “Good Laboratory Rules practice”, approved by order of the Ministry of Health of the Russian Federation No. 199n of 04/01/2016. Primary cultures of rat brain cortical neurons were prepared from the cortex of one- or two-day old Wistar rats. The rats were anesthetized, decapitated, and the cortex was removed and separated from the meninges. The extracted tissues were washed in a Ca^2+^- and Mg^2+^-free Hanks solution, crushed, and placed in a papain solution for 15 min at 36 °C, washed with standard Hanks solution with phenol red and Minimal Essential Medium (MEM) culture medium, and dispersed in fresh MEM. A homogeneous suspension was precipitated two times in a centrifuge at 200 g for 5 min. The precipitated cells were resuspended to a concentration of 10^6^ cells/ml in neurobasal medium (NBM), supplemented with B-27 Supplement, GlutaMAX, and penicillin/streptomycin. The suspension (200 μl) was transferred onto coverslips attached to the wells of 35 mm plastic Petri dishes (MatTek, Ashland, MA, USA) or a volume of 400 μl into each well of 24-well plastic plates (Corning costar). The glass dishes and plates were pre-coated with 10 mg/ml of polyethyleneimine for 30 min. After one hour, 1.5 ml of NBM, containing 2% B-27Supplement, 1% antibiotic-antimycotic, and 1% GlutaMAX, was added. The cells were kept in an incubator at 37 °C, 95% air + 5% CO_2_, and a relative humidity of 100%. Cytosine arabinoside (AraC, 5 μM) was added to the medium for two or three days to prevent the proliferation of glial cells and obtain cultures with a percentage of neurons of more than 90%. Every three days, the cells were fed by replacing 1/3 of the old medium with new medium. Cultures were used in experiments 10–12 days after plating (10–12 days in culture, DIV). Before every experiment, bottoms with the cells and plates were washed ten times out of the B27 supplement with a buffer containing: (mM): 135 NaCl, 5 KCl, 2 CaCl_2_, 1 MgCl_2_, 20 HEPES, 5 D-glucose; pH 7.4. Then, the cells were kept in this buffer for one hour before every experiment.

**Phenotyping of cell cultures**

The culture was fixed with 4% PFA for 15 minutes, washed with PBS, permeabilized with ice-cold methanol for 15 minutes on ice, and washed three times with PBS. The non-specific antibody binding was blocked with 2% BSA in PBS with the addition of 0.05% Tween 20 (PBS-T) for 20 minutes at room temperature. Specific primary antibodies to bIIITubulin (PA5-85639) and GFAP proteins (OPA1-06100) were added in a solution of 2% BSA in PBS-T at 1:1000 and 1:100 dilutions, respectively, and incubated overnight at +4 °C. After washing three times with PBS, secondary antibodies conjugated with FITC (QL230437) and AlexaFuor 594 (A-11005) were added in a solution of 2% BSA in PBS-T at a 1:100 dilution and incubated for 30 minutes at room temperature in the dark. After washing three times with PBS, a DAPI solution (D3571) was added to stain the nuclei. Fluorescence microscopy was performed on Cytation 3 Cell Imaging Multi-Mode Reader from BioTek and background fluorescence was subtracted. Cell counts were performed using CellProfiler v. 3.1.8 and classification CellProfiler Analyst v. 2.2.1. The percentage of cells expressing each marker in each biological replicate was calculated relative to the total number of cells with live morphology (DAPI-positive cells and bIIITub or GFAP positive cells). Data are presented as mean ± SEM for ten independent cultures. Neurons (bIIITub positive and GFAP negative cells) account for 90.5 ± 13.8 % of total cells and glial cells (GFAP positive and bIIITub negative cells) account for 9.5 ± 5.4 %. Figure 1S shows representative images of the culture of rat cortical cells stained with fluorescent markers on DAPI, bIIITub, and GFAP.

**Measurement of [Ca^2+^]_i_ and ΔΨ_m_**

For [Ca^2+^]_i_ measurements, cortical neurons were loaded with a low affinity Ca^2+^indicator, Fura-FF (2 μM), in the form of acetoxymethyl esters, for 60 min at 37 °C. A non-ionic detergent, PluronicF-127 (0.02%; Molecular Probes, USA), was added to facilitate the penetration of the Fura-FF into the cells. Fura-FF fluorescence was excited alternately at 340 and 380 nm and recorded at 525 nm (dichroic mirror 500 nm). For simultaneous measurements of [Ca^2+^]_i_ and the ΔΨ_m_, cells were loaded for the last 15 min of the “Fura-FF loading period” in buffer at 37 °C with 2.5 μg/ml of Rhodamine 123 (Rh123). Rh123 fluorescence was excited and recorded at 485 and 525 nm, respectively. Accumulation of Rh123 in polarized mitochondria quenches the fluorescent signal. In response to depolarization, the fluorescence is dequenched [5]. The measurements were performed at 25–27 °С in a medium containing (mM): 135 NaCl, 5 KCl, 2 CaCl_2_, 1 MgCl_2_, 20 HEPES, 5 D-glucose; pH 7.4. Glutamate 100 μM was added in Mg^2+^ free, 10 μM glycine containing medium and the cells were then exposured at 37 °C for a period of 30 min. In Ca^2+^-free buffers, CaCl_2_ was replaced with 0.1 mM EGTA and 2 mM MgCl_2_. To examine mitochondrial Ca^2+^ accumulation during glutamate exposure, mitochondria were completely depolarized by adding 1μM of carbonyl-p- (trifluoromethoxy) phenylhydrazone (FCCP). Finally, 1 μM of Ionomycine (Iono) was added to calibrate the maximal response of Fura-FF to a high rise in [Ca^2+^]_i_. Fluorescence measurements were performed using a fluorescence imaging system, which consisted of an Olympus IX-71 inverted microscope equipped with a 175 W xenon lamp, 20 × fluorite objective, a Sutter Lambda 10-2 illumination system (Sutter Instruments, Novato, CA, USA), and a CoolSNAP HQ2 CCD camera operated by the computer program MetaFluor (Molecular Device, San Jose, CA, USA).

**Measurement of phosphoproteins IRβ pY^1150/1151^, Akt pS^473^, mTOR pS^2448^, and GSK3β pS^9^**

For measurements of phosphoproteins, rat cortical neurons were exposed to 100 µM glutamate or none in Mg^2+^ free, 10 μM glycine containing medium at 37 °C for a period of 30 min. Then, the medium was substituted with buffer containing 100 nM insulin, 130 mM NaCl, 5.4 mM KCl, 5 mM D-glucose, 20 mM HEPES, and pH 7.4 and cells were incubated at 37 °C for a period of 15 min. Cell were lysed, levels of phosphoproteins IRβ p^Y1150/1151^, Akt pS^473^, mTOR pS^2448^, and GSK3β pS^9^ in lysates were measured with MILLIPLEX® MAP Akt/mTOR phosphoprotein panel (EMD Millipore Corp. MA, USA; cat. no. 48-611MAG) and levels of total proteins IRβ, Akt, mTOR, and GSK3β in the lysates were measured with MILLIPLEX® MAP Akt/mTOR total protein panel (EMD Millipore Corp. MA, USA; cat. no. 48-612MAG) using MAGPIX® analyzer (Merck Millipore, MA, USA) in accordance with manufacturer's instructions. The obtained phosphoprotein values of IRβ p^Y1150/1151^, Akt pS^473^, mTOR pS^2448^, and GSK3β pS^9^ were normalized to total protein levels of IRβ, Akt, mTOR, and GSK3β, respectively, and expressed as a percentage of insulin treated cells.


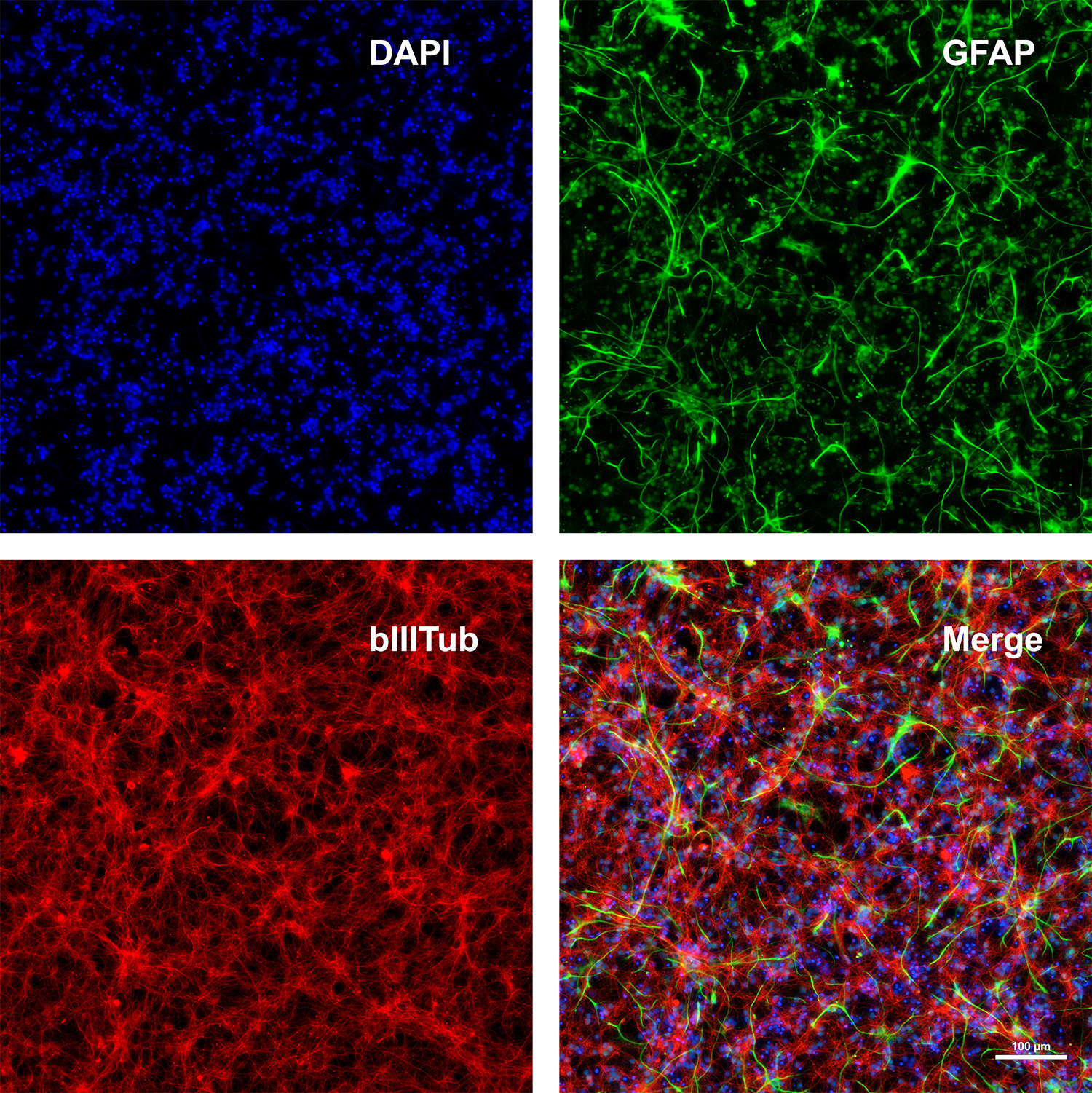


**Figure 1S. Representative images of fluorescence staining of cell culture on (a) DAPI, (b) GFAP, (c) bIIITubulin, and (d) Merge, scalebar 100 µm. (a)**

**Table S1.** Raw data of phosphoproteins measurements in cell lysates.

| Phosphoprotein | Groups | | | |  |
| --- | --- | --- | --- | --- | --- |
|  | Control | Insulin | Glutamate | Glutamate + Insulin | |
| IRβ pY^1150/1151^ / IRβ, % | 46,96  44,23  35,77  42,32  37,41  43,69 | 103,71  78,57  72,46  123,99  135,88  85,39 | 42,05  25,32  30,75  35,85  36,69  15,47 | 46,11  48,19  47,55  76,76  39,12  54,71 |  |
| Akt pS^473^ / Akt, % | 23,81  30,53  24,82  25,71  26,60  25,89 | 101,72  89,97  107,76  112,13  99,12  89,30 | 11,49  10,69  11,10  10,76  9,22  9,18 | 28,06  28,22  26,13  32,67  25,02  26,17 |  |
| mTOR pS^2448^ / mTOR, % | 70,43  47,60  55,76  68,17  56,29  68,11 | 137,53  78,19  94,11  102,65  84,80  102,18 | 46,61  42,44  30,62  42,57  48,22  45,20 | 58,99  47,58  32,98  64,13  60,52  70,36 |  |
| GSK3β pS^9^ / GSK3βm, % | 97,37  79,61  64,48  63,82  78,29  75,66 | 110,90  73,09  83,25  97,47  104,61  130,68 | 105,69  75,55  55,15  96,39  70,50  48,63 | 59,66  57,97  52,66  94,49  55,47  52,33 |  |

**Table S2.** Results of statistical analysis.

| IRβ pY^1150/1151^ / IRβ, % | | | | |
| --- | --- | --- | --- | --- |
|  | C | I | G | G+I |
| C |  |  |  |  |
| I | **** |  | **** |  |
| G | ns |  |  | ns |
| G+I | ns | *** |  |  |
| Akt pS^473^ / Akt, % | | | | |
| C |  |  |  |  |
| I | **** |  | **** |  |
| G | *** |  |  | **** |
| G+I | ns | **** |  |  |
| mTOR pS^2448^ / mTOR, % | | | | |
| C |  |  |  |  |
| I | *** |  | **** |  |
| G | ns |  |  | ns |
| G+I | ns | **** |  |  |
| GSK3β pS^9^ / GSK3β, % | | | | |
| C |  |  |  |  |
| I | ns |  | ns |  |
| G | ns |  |  | ns |
| G+I | ns | ** |  |  |

**P < 0.01, ***P < 0.001, ****P < 0.0001, ns denotes P > 0.05. One-way ANOVA followed by Tukey’s test for multiple comparisons between groups.
